# Supplementary material for: Mapping of Quantitative Trait Loci Controlling Egg-Quality and -Production Traits in Japanese Quail (Coturnix japonica) Using Restriction-Site Associated DNA Sequencing
Source: Genes (Basel). 2021 May 13;12(5):735. doi: 10.3390/genes12050735 (PMC8153160; doi:10.3390/genes12050735)
Supplement: Supplementary file 1 [file genes-12-00735-s001.zip › genes-1172563-supplementary.pdf]

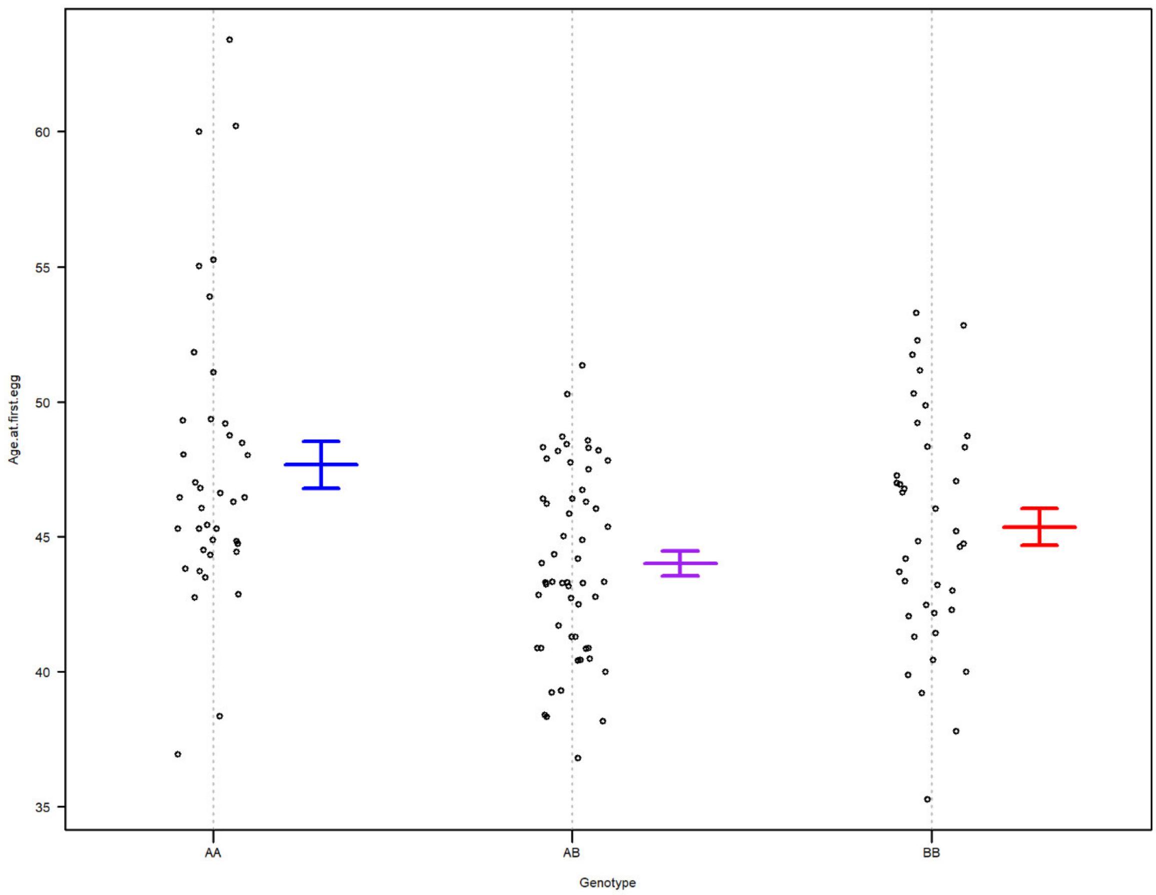

Figure S1. Plot of the phenotype against the genotypes for age at first egg QTL detected on chromosome 2.

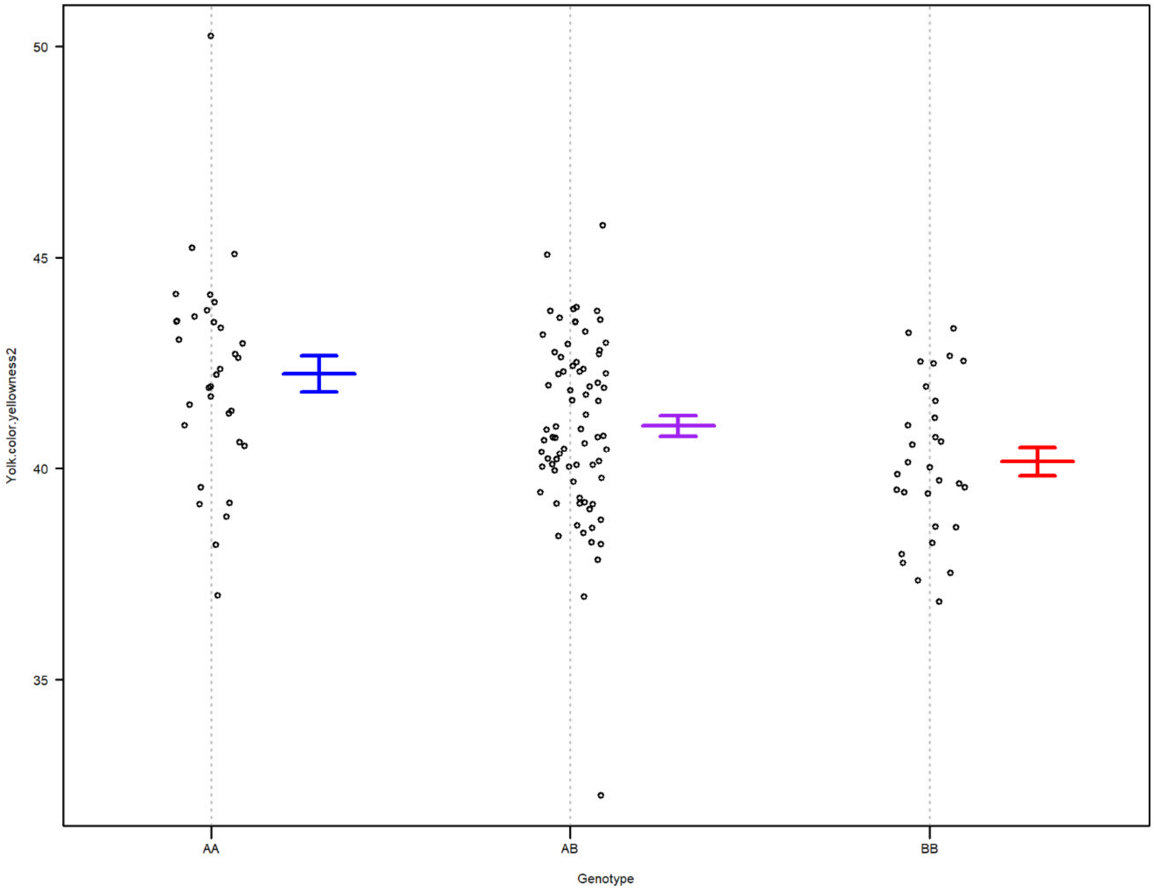

Figure S2. Plot of the phenotype against the genotypes for yolk color-yellowness2 QTL detected on chromosome 2.

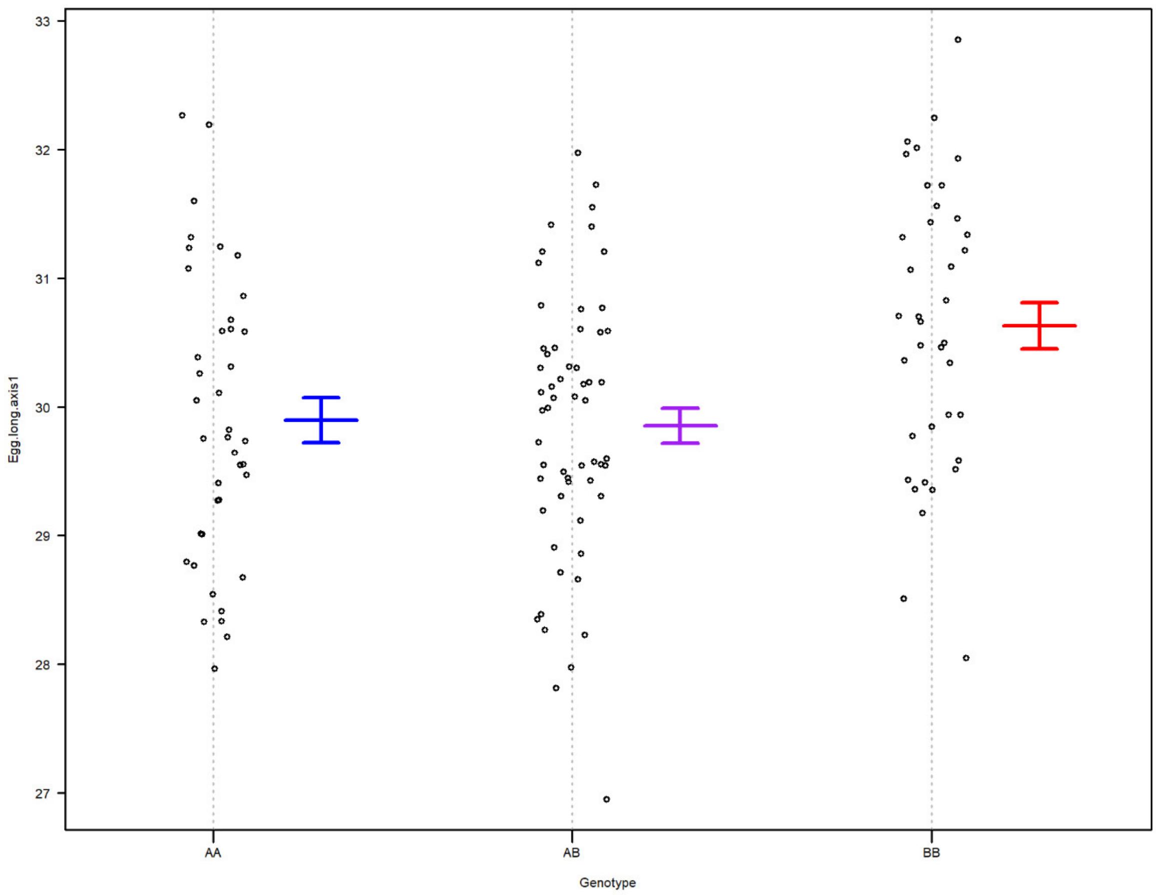

Figure S3. Plot of the phenotype against the genotypes for egg long axis1 QTL detected on chromosome 4.

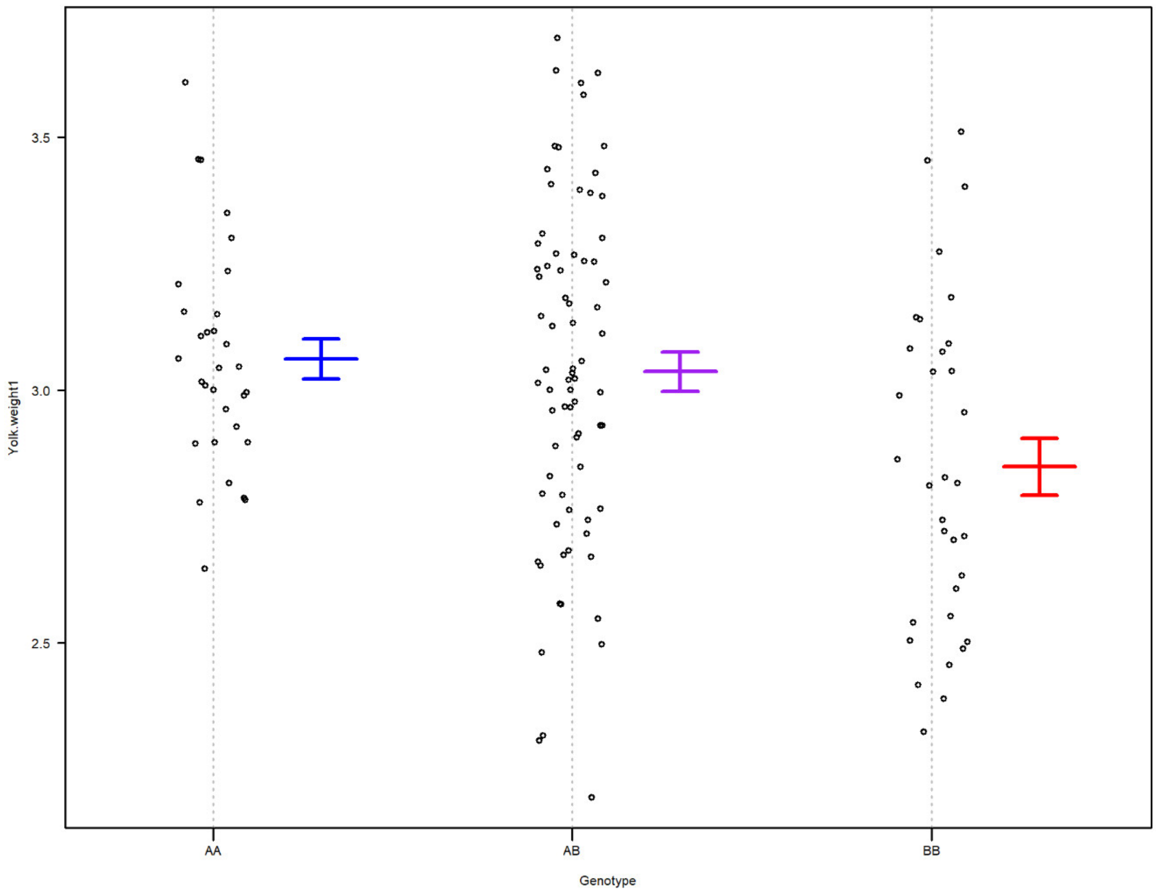

Figure S4. Plot of the phenotype against the genotypes for yolk weight1 QTL detected on chromosome 6.

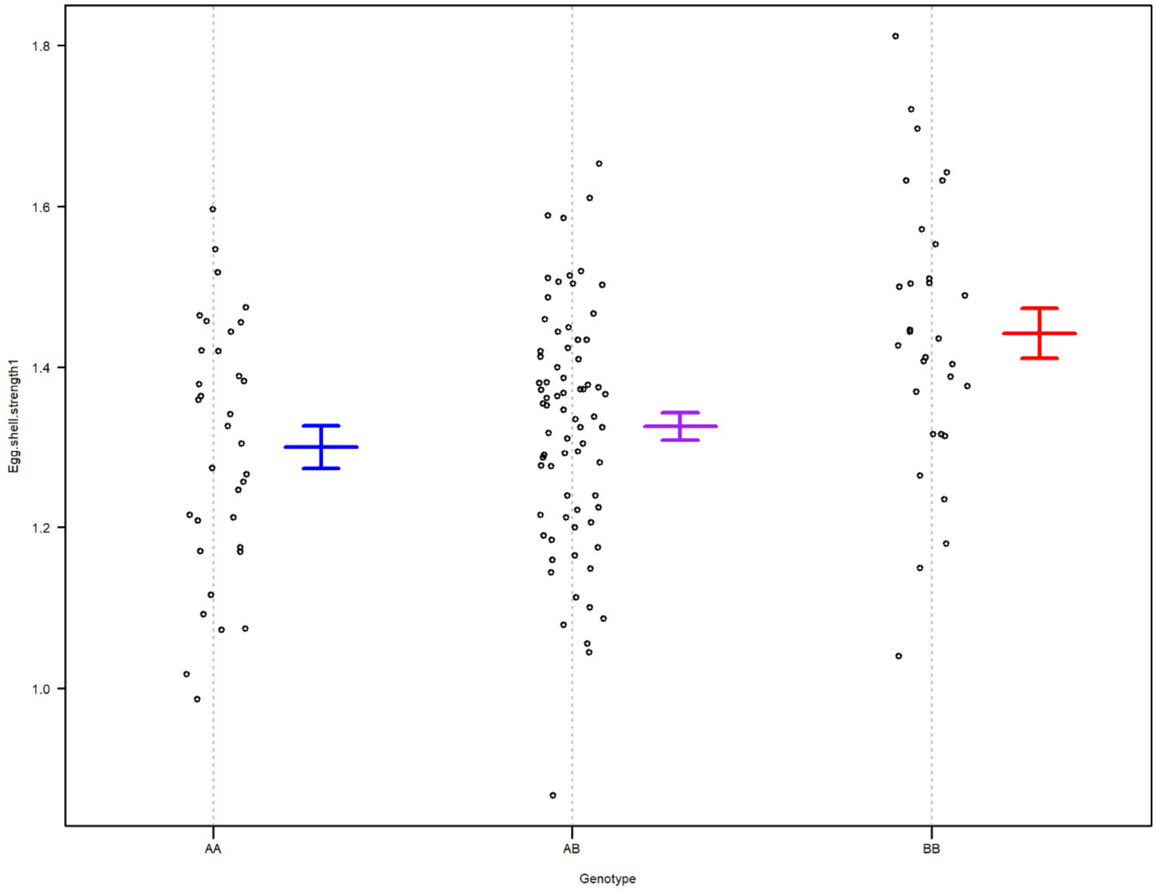

Figure S5. Plot of the phenotype against the genotypes for egg shell strength1 QTL detected on chromosome 6.

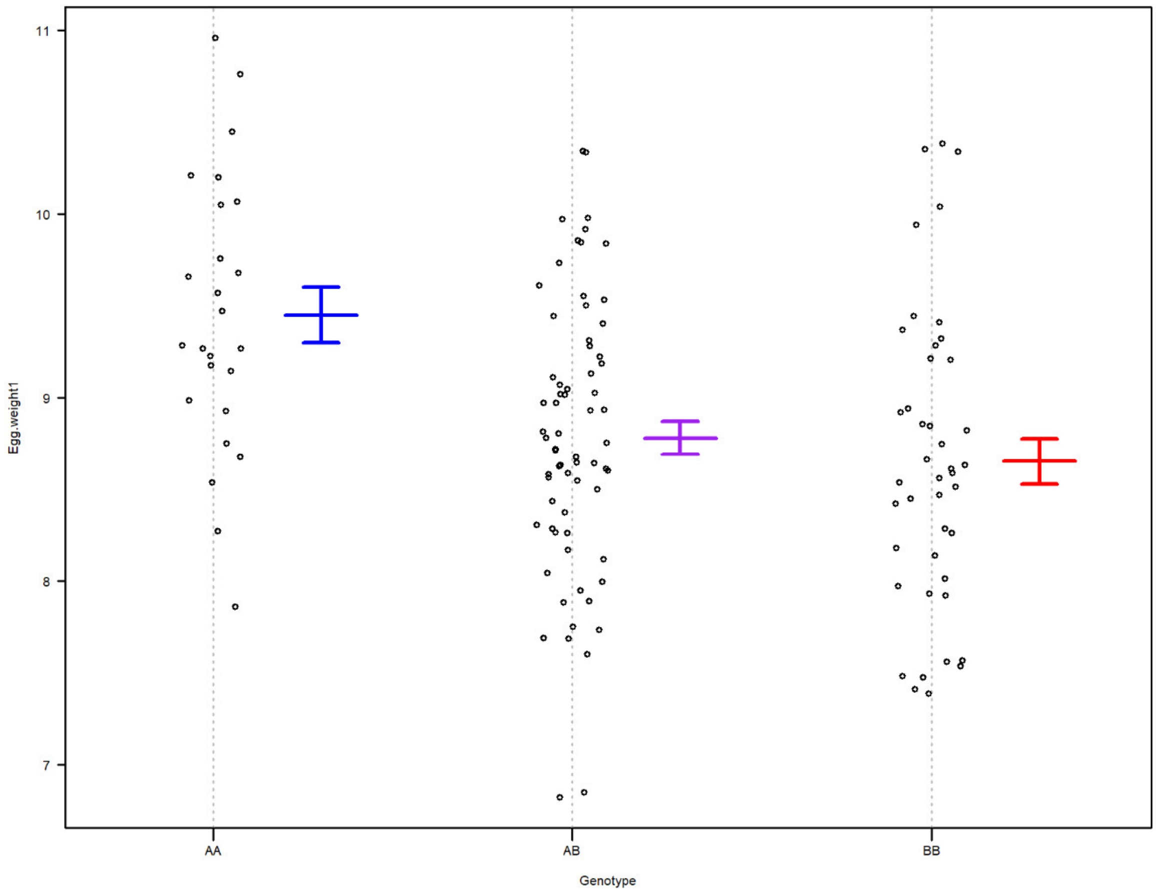

Figure S6. Plot of the phenotype against the genotypes for egg weight1 QTL detected on chromosome 10.

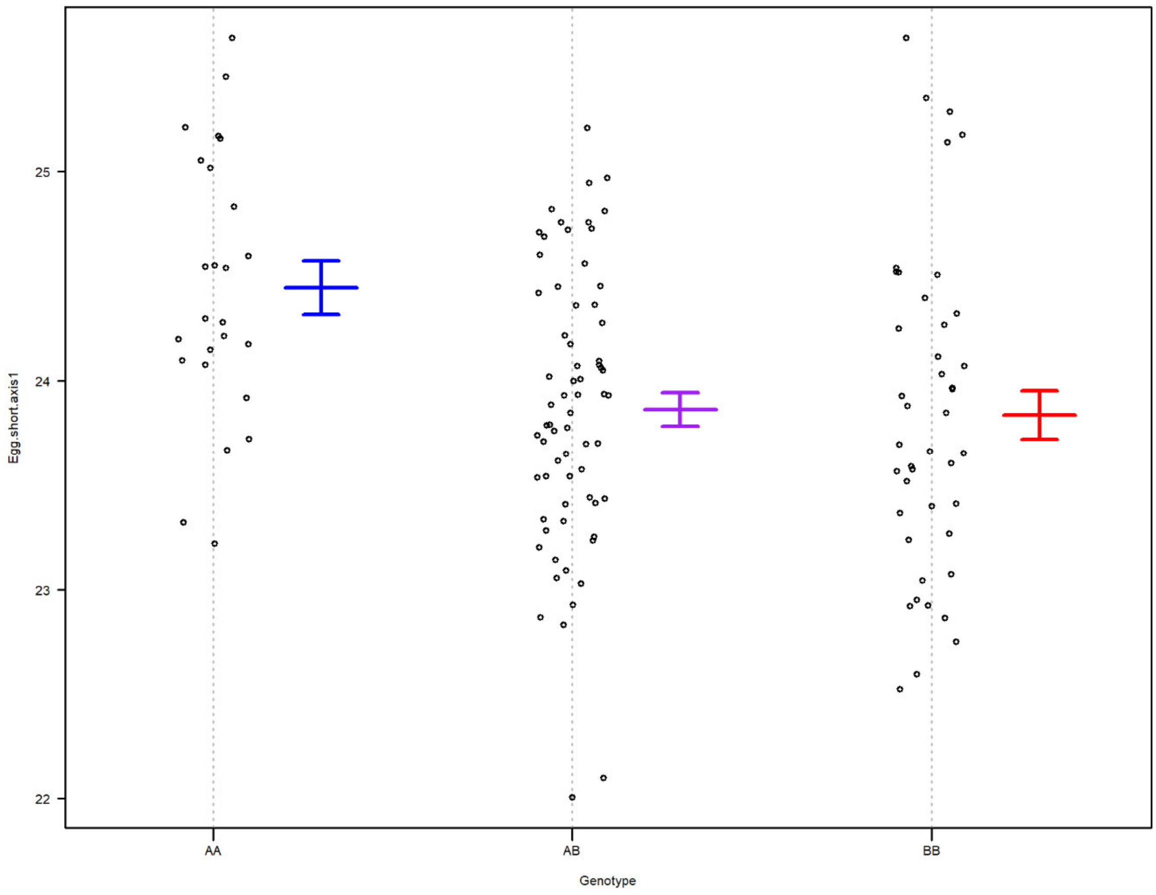

Figure S7. Plot of the phenotype against the genotypes for egg short axis$_1$ QTL detected on chromosome 10.

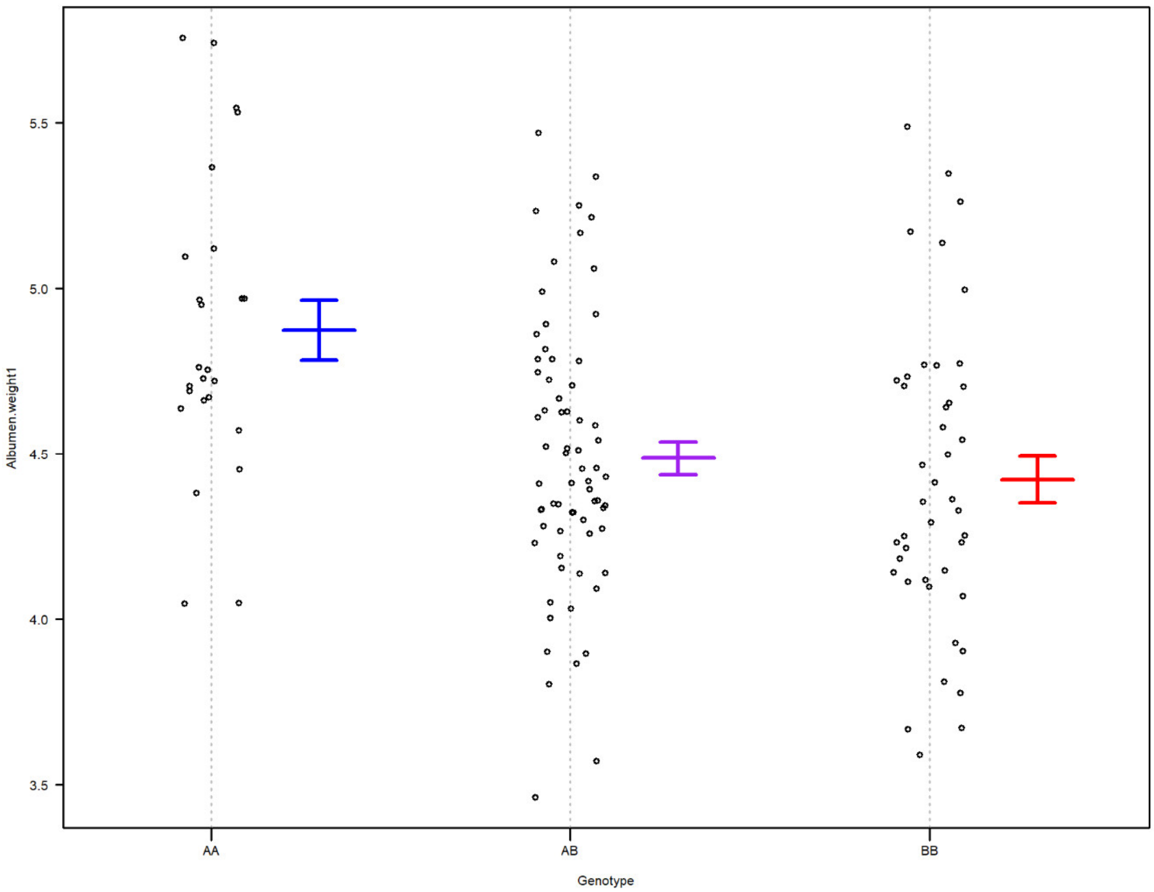

Figure S8. Plot of the phenotype against the genotypes for albumen $weight_1$ QTL detected on chromosome 10.

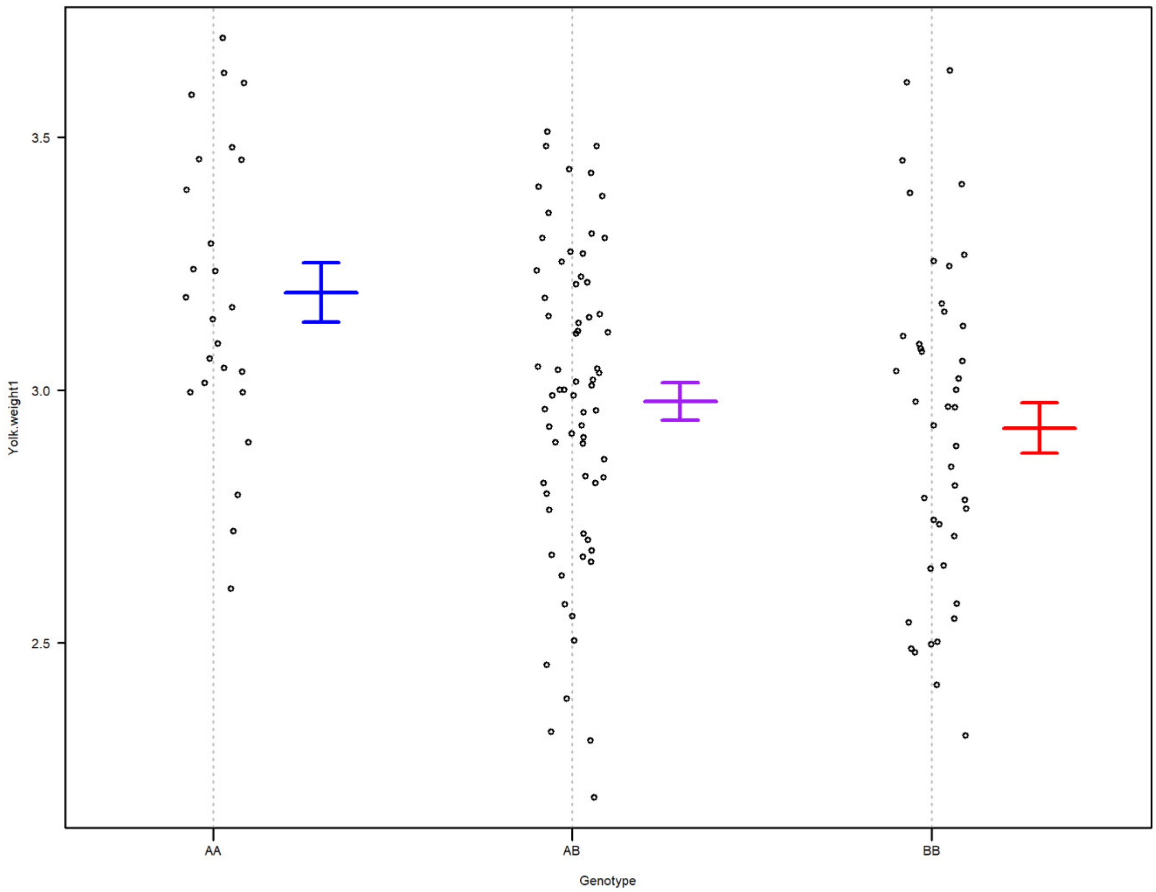

Figure S9. Plot of the phenotype against the genotypes for yolk weight$_1$ QTL detected on chromosome 10.

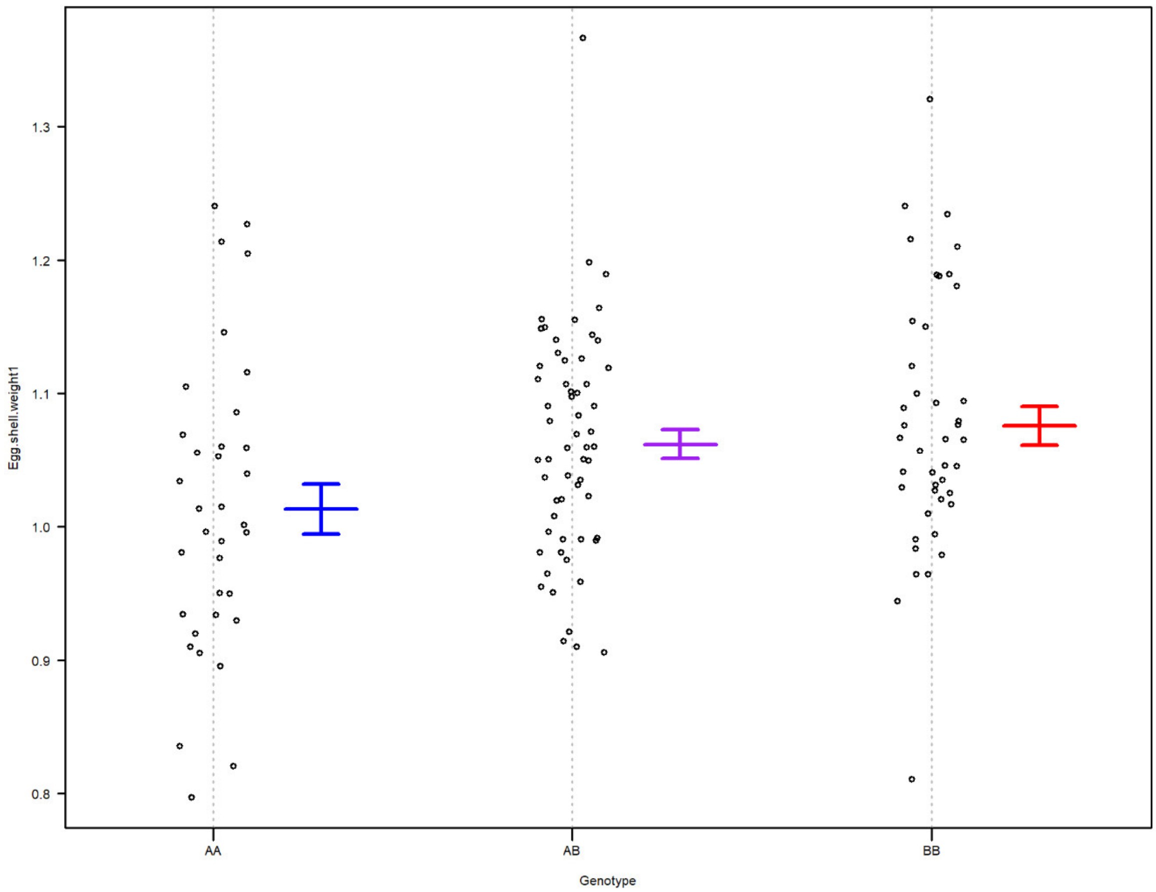

Figure S10. Plot of the phenotype against the genotypes for egg shell weight1 QTL detected on chromosome 12.

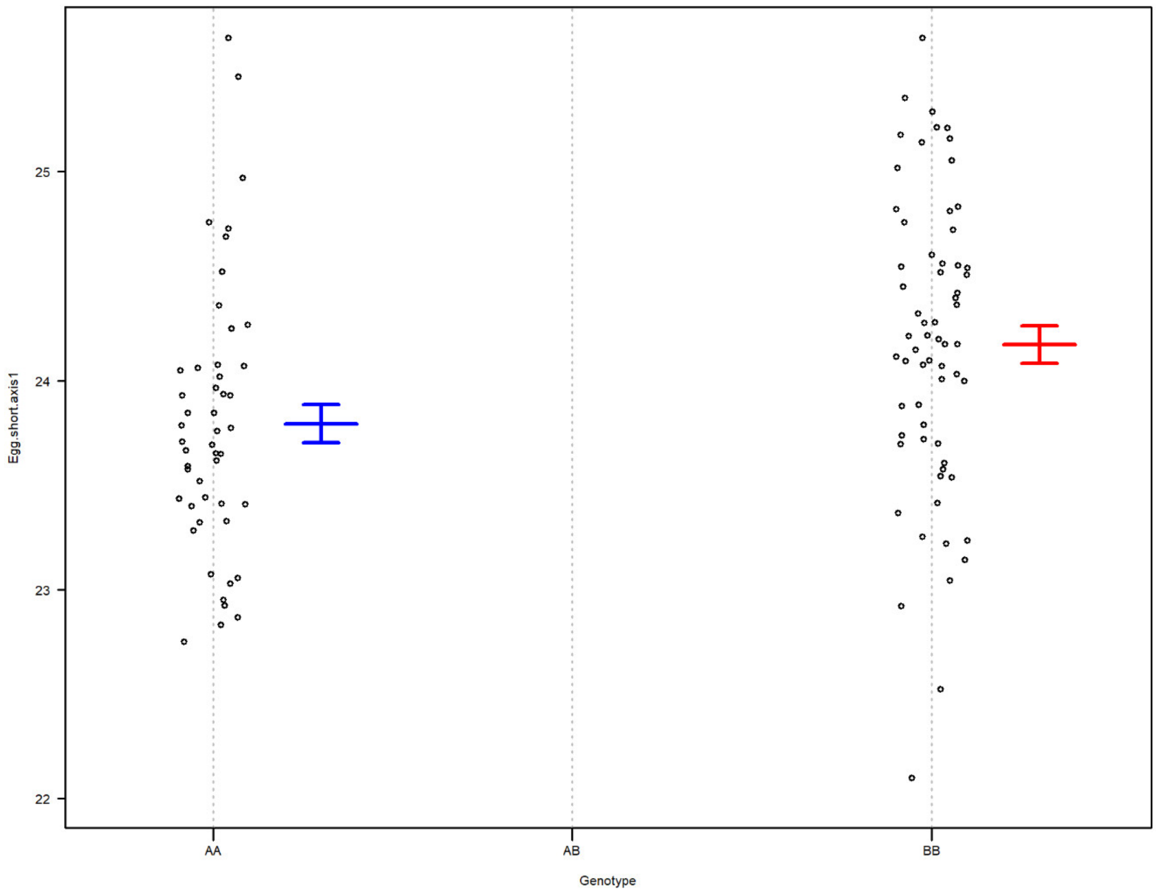

Figure S11. Plot of the phenotype against the genotypes for egg short axis$_1$ QTL detected on Z chromosome.

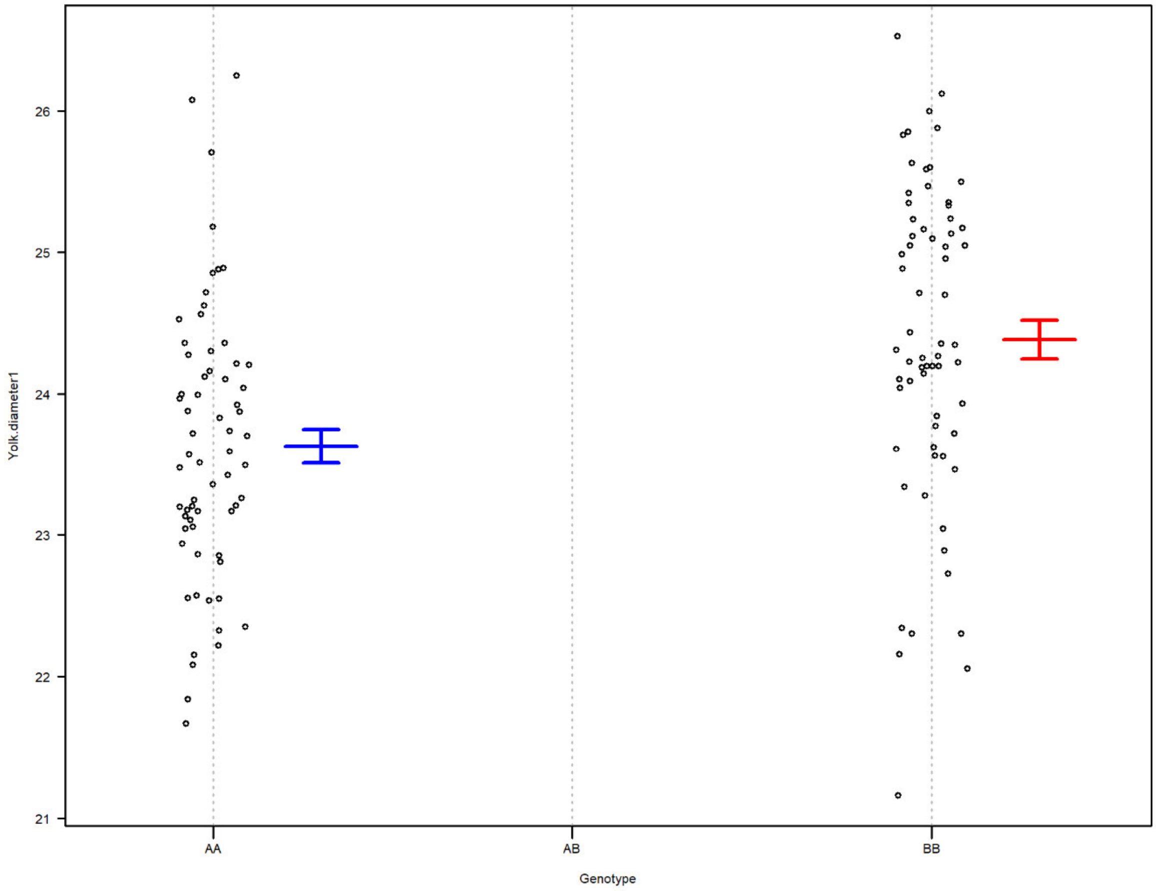

Figure S12. Plot of the phenotype against the genotypes for yolk diameter$_1$ QTL detected on Z chromosome.
